# Post-COVID Multisystem Inflammatory Syndrome in Children with Kawasaki-like Features: A Scoping Review of Middle Eastern Evidence

**DOI:** 10.3390/medicina62081600

**Published:** 2026-08-20

**Authors:** Hajira Wase, Mahek Nichlani, Ayesha Shaikh, Snigda Mahanti, Subhranshu Sekhar Kar, Shria Sadhu, Rajani Dube, Manjunatha Goud Bellary Kuruba

**Affiliations:** 1 RAK Medical and Health Sciences University, Ras-Al Khaimah 12973, United Arab Emirates; hajira.22901110@rakmhsu.ac.ae (H.W.); mahek.nichlani@gmail.com (M.N.); ayesha.23901013@rakmhsu.ac.ae (A.S.); snigda.22901056@rakmhsu.ac.ae (S.M.); 2 Pediatrics, RAK Medical and Health Sciences University, Ras-Al Khaimah 12973, United Arab Emirates; subhranshu.kar@rakmhsu.ac.ae; 3 Obstetrics and Gynecology, RAK Medical and Health Sciences University, Ras-Al Khaimah 12973, United Arab Emirates; rajani.dube@rakmhsu.ac.ae; 4 Biochemistry, RAK Medical and Health Sciences University, Ras-Al Khaimah 12973, United Arab Emirates; manjunatha@rakmhsu.ac.ae

**Keywords:** multisystem inflammatory syndrome in children, MIS-C, Kawasaki disease, SARS-CoV-2, Middle East, myocarditis, coronary arteries, scoping review

## Abstract

*Background*: Multisystem inflammatory syndrome in children (MIS-C) was recognized in 2020 as a post-infectious hyperinflammatory condition occurring 2–6 weeks after SARS-CoV-2 infection. While large studies from Europe and North America have described its clinical spectrum and outcomes, evidence from the Middle East remains limited. MIS-C shares features with Kawasaki disease (KD), including persistent fever, mucocutaneous manifestations, and cardiac involvement, but differs in age distribution, gastrointestinal involvement, and patterns of cardiac injury. *Objective*: To map the reported Middle Eastern evidence on the clinical and laboratory phenotype, Kawasaki-like manifestations, cardiovascular involvement, management, short-term outcomes, and evidence gaps in children with MIS-C. *Methods*: A scoping review was conducted using the Arksey and O’Malley framework, guided by Joanna Briggs Institute methodology and reported according to PRISMA-ScR. PubMed/MEDLINE, Scopus, Web of Science, and the WHO COVID-19 database were searched for studies published from 1 December 2020 through 30 September 2025. Two reviewers independently screened records in Rayyan and charted data using standardized forms. Findings were synthesized descriptively; numerical ranges reflect values reported across selected contributing studies and are not pooled regional estimates. *Results*: Twenty-seven original clinical studies met the eligibility criteria, including case reports, case series, and observational cohorts from the UAE, Saudi Arabia, Qatar, Jordan, and multinational Middle Eastern collaborations. Across the included reports, affected children were generally older than the typical classic Kawasaki disease population. Gastrointestinal symptoms were commonly reported, and myocardial dysfunction was reported more frequently than coronary artery abnormalities. Most patients received intravenous immunoglobulin, often combined with corticosteroids. Short-term outcomes were generally favorable, but the evidence was heterogeneous and follow-up was limited. No meta-analysis was performed. *Conclusions*: The available Middle Eastern literature suggests that MIS-C commonly presents in school-aged children with gastrointestinal, mucocutaneous, and myocardial involvement. These patterns are descriptive rather than definitive because the evidence is predominantly retrospective, geographically uneven, and methodologically heterogeneous. The principal contribution of this review is to map the regional literature and identify priorities for prospective, standardized, multicenter studies and long-term follow-up.

## 1. Introduction

Since the start of the COVID-19 pandemic, multisystem inflammatory syndrome in children (MIS-C) has been identified early as a severe complication of SARS-CoV-2 in children. The children who developed this syndrome presented with a fever lasting 2–6 weeks after an initial SARS-CoV-2 infection, which was observed in the U.K. and Italy in 2020 [1,2]. Distinct from acute COVID-19, this syndrome was formally termed “multisystem inflammatory syndrome in children” (MIS-C) by the World Health Organization (WHO) and Centers for Disease Control and Prevention (CDC) and “pediatric inflammatory multisystem syndrome temporally associated with SARS-CoV-2” (PIMS-TS) in the United Kingdom [1]. It rapidly became recognized as one of the most significant pediatric complications of the pandemic.

Unlike acute COVID-19, MIS-C is not a consequence of direct viral tissue injury. Rather, it is understood to reflect a dysregulated post-infectious immune response, possibly triggered by retained viral antigens or cross-reactive immune activation, though the precise immunopathogenic mechanisms remain an area of active investigation [3].

The clinical syndrome is to a large extent overlapping with Kawasaki disease (KD), a yet-unknown-etiology acute vasculitis mainly involving small and medium-sized vessels in young children. Shared features include persistent fever, conjunctival injection, polymorphous rash, oral mucosal changes, and extremity edema, and both may involve the coronary arteries and myocardium, which is sometimes difficult to distinguish at presentation [1,3,4].

In contrast to KD, which predominantly affects children under five years, MIS-C typically presents in school-aged children and adolescents. Gastrointestinal symptoms, particularly abdominal pain and vomiting, are strikingly frequent in MIS-C and often dominate the early presentation, while marked lymphopenia, coagulopathy, and elevated cardiac biomarkers such as BNP/NT-proBNP and troponin are more pronounced than in classic KD [5].

Despite this clinical overlap, MIS-C and KD differ in ways that have diagnostic and therapeutic implications. Comparative studies and immunologic profiling suggest MIS-C and KD are overlapping but distinct syndromes, with MIS-C showing deeper lymphopenia, more frequent myocardial dysfunction, and different cytokine signatures [2].

International cohorts from North America and Europe have established the clinical epidemiology of MIS-C, reporting in-hospital mortality of approximately 1–2%, PICU admission in up to 50% of cases, and favorable recovery of cardiac function with prompt immunomodulatory therapy. However, these large series were conducted in specific healthcare and demographic contexts that may not reflect the Middle Eastern experience [1,2].

Published reports from the UAE, Qatar, Saudi Arabia, Jordan, and multinational regional collaborations confirm that MIS-C occurs in Middle Eastern children, but the literature is geographically uneven and consists largely of retrospective cohorts, case series, and case reports with variable follow-up. A structured mapping of the reported regional evidence, with explicit attention to Kawasaki-like manifestations, cardiovascular outcomes, management, and knowledge gaps, has not previously been undertaken [6].

Regional epidemiology warrants study because populations, healthcare access, referral pathways, and treatment availability differ across settings. Limited regional genetic studies have identified candidate immune-related variants, but the current evidence is insufficient to establish a population-wide genetic distinction or a causal difference from European or North American populations.

Applying Western MIS-C management frameworks without understanding these contextual factors risks both under- and overtreatment [4,5,6].

Given the heterogeneity and rapid growth of this literature, a scoping review, which aims to map and characterize evidence rather than synthesize it quantitatively, is the most appropriate design at this stage [3,4,5].

Using the PCC (Population-Concept-Context) framework, the review asked the following question: What evidence has been reported from Middle Eastern clinical settings regarding the phenotype, Kawasaki-like manifestations, cardiovascular involvement, management, short-term outcomes, and remaining knowledge gaps of MIS-C in children and adolescents? Kawasaki-like features were selected as a conceptual anchor because mucocutaneous and coronary manifestations overlap with classic Kawasaki disease, whereas the age distribution, gastrointestinal burden, shock, and myocardial dysfunction of MIS-C may differ. The purpose was evidence mapping and gap identification rather than estimation of pooled effects [7,8,9,10].

## 2. Methods

### 2.1. Review Design

We conducted a scoping review guided by the Arksey and O’Malley framework and Joanna Briggs Institute (JBI) methodological guidance and reported it in accordance with the PRISMA Extension for Scoping Reviews (PRISMA-ScR). The scoping-review design was selected to map heterogeneous regional evidence and identify gaps; it was not intended to estimate pooled prevalence or treatment effects. The PRISMA-ScR checklist is provided in the Supplementary Materials. The review protocol was not prospectively registered.

### 2.2. Conceptual Framework (PCC)

The eligibility criteria were developed according to the Population–Concept–Context (PCC) framework:

- **Population**: Children and adolescents younger than 18 years diagnosed with MIS-C or PIMS-TS according to WHO, CDC, RCPCH, or clearly stated author-defined criteria that required evidence of SARS-CoV-2 infection or exposure [1,2,11].
- **Concept**: MIS-C/PIMS-TS with reported Kawasaki-like involvement. For this review, Kawasaki-like features were operationalized as an explicit Kawasaki-like phenotype or reporting of at least one recognized Kawasaki-related domain: bilateral non-purulent conjunctival injection; oral or lip changes; polymorphous rash; extremity changes; cervical lymphadenopathy; or coronary artery dilatation/aneurysm. Studies also had to report at least one clinical, laboratory, cardiovascular, management, or outcome domain relevant to the review question.
- **Context**: Clinical settings in Middle Eastern countries. Because the retrieved evidence was concentrated in the Gulf and a small number of neighboring settings, country-level coverage is reported transparently and absence of evidence from other Middle Eastern countries is not interpreted as absence of disease [12,13].

### 2.3. Eligibility Criteria

#### 2.3.1. Inclusion Criteria

- Original clinical studies, including case reports, case series, cohort studies, cross-sectional studies, and multicenter observational studies, involving children or adolescents with MIS-C/PIMS-TS in Middle Eastern clinical settings.
- Studies reporting an explicit Kawasaki-like phenotype or at least one prespecified Kawasaki-related domain, together with extractable information on clinical presentation, laboratory findings, cardiovascular manifestations, management, or outcomes.
- Articles published from 1 December 2020 through 30 September 2025.
- Articles published in any language, provided that sufficient clinical information could be translated or extracted.

#### 2.3.2. Exclusion Criteria

- Studies conducted exclusively in adults aged 18 years or older.
- Studies not involving patients from Middle Eastern countries.
- Studies focused exclusively on classic Kawasaki disease without an association with SARS-CoV-2 or MIS-C.
- Editorials, commentaries, narrative or systematic reviews, and clinical guidelines without original clinical data.
- Non-clinical laboratory studies and animal studies.
- Publications with insufficient extractable clinical data or duplicate reporting of the same study population.

### 2.4. Information Sources and Search Strategy

Four bibliographic databases were searched: PubMed/MEDLINE, Scopus, Web of Science, and the WHO COVID-19 database. The search covered publications from 1 December 2020 through 30 September 2025. The strategy combined controlled vocabulary, where available, and free-text terms for MIS-C/PIMS-TS, SARS-CoV-2, Kawasaki-like manifestations, and Middle Eastern countries; syntax was adapted for each database. Reference-list and citation searching of relevant records yielded 51 additional records. No language restriction was applied; non-English reports were considered when adequate information could be translated and extracted.

The core search logic was: (“MIS-C” OR “multisystem inflammatory syndrome in children” OR “PIMS-TS” OR “pediatric inflammatory multisystem syndrome”) AND (SARS-CoV-2 OR COVID-19) AND (Kawasaki OR “Kawasaki-like” OR mucocutaneous OR coronary) AND (“Middle East” OR names of individual Middle Eastern countries). Database-specific field tags and controlled vocabulary were adapted to each source. Records from the four databases were exported into a combined dataset before deduplication and screening; the retained documented database total was 1578 records.

The search did not systematically cover Embase, regional indexes, grey literature, or conference proceedings; therefore, incomplete retrieval and regional publication bias remain possible despite the inclusion of Scopus and Web of Science.

### 2.5. Study Selection

The four database searches identified 1578 records, and reference-list/citation searching identified 51 additional records, giving 1629 records before deduplication. After 448 duplicates were removed, 1181 unique records underwent title and abstract screening.

Two reviewers independently screened titles and abstracts against the predefined criteria using Rayyan, a web-based platform for systematic and scoping review screening. Of 1181 screened records, 733 were excluded. All 448 reports sought for full-text assessment were retrieved. After full-text review, 421 reports were excluded (not from a Middle Eastern setting, *n* = 198; adult or otherwise incorrect population, *n* = 101; no extractable clinical data, *n* = 79; other eligibility criteria not met, n = 43), leaving 27 original clinical studies represented by 27 reports.

**Figure 1:**
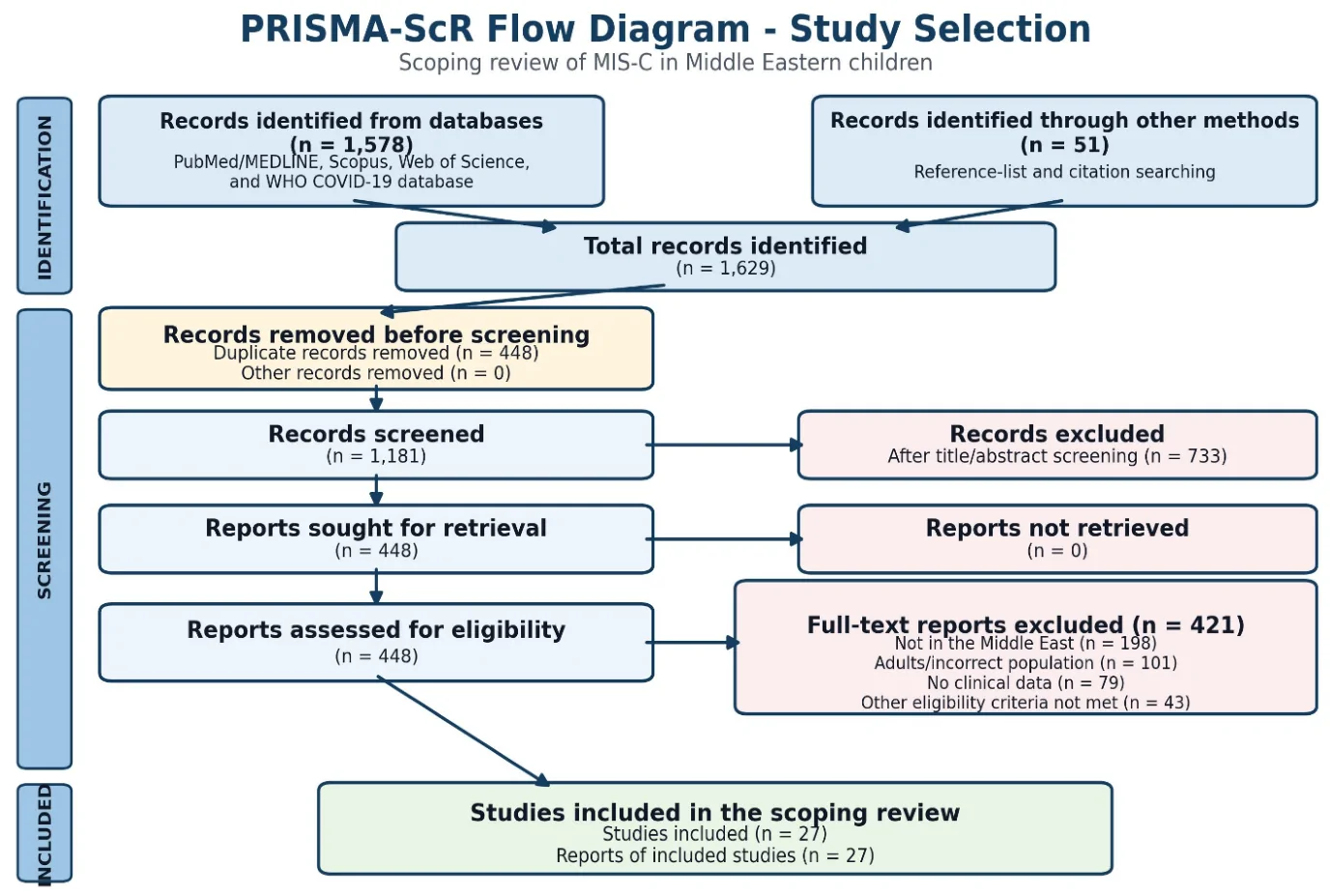
PRISMA-ScR flow diagram documenting the study selection process for the scoping review on MIS-C in Middle Eastern children (*n* = 27 studies included). Adapted from PRISMA 2020 flow diagram.

Disagreements were resolved by discussion, with consultation of a third reviewer when consensus could not be reached. The complete selection process is shown in Figure 1. Potentially overlapping study populations were considered during interpretation; however, incomplete reporting of institutions, recruitment periods, and sample characteristics limited definitive assessment. No patient-level datasets or study denominators were combined quantitatively.

### 2.6. Data Charting

Extracted data for the variables of interest comprised the following:

**Description of study:** country of origin, setting, study design, time period covered by the study, and total number of subjects.

**Demographic data:** age, sex ratio.

**Symptoms observed:** fever, mucocutaneous symptoms, gastrointestinal involvement, respiratory involvement, neurologic involvement, and other symptoms.

Tests: inflammation (CRP, ferritin, ESR), blood, coagulation markers (D-dimer), and heart markers (troponin, BNP/NT-proBNP).

**Heart-related information:** left ventricular ejection fraction, valve regurgitation, coronary z-scores, arrhythmias, PICU admission, need for vasopressors.

**Management:** IVIG, steroids, biologics (anakinra, tocilizumab), anticoagulants or antiplatelets, and hemodynamic management.

**Outcomes:** mortality in the hospital, length of stay in the hospital, recovery of ventricular function, resolving coronary involvement, and duration of follow-up.

Two reviewers independently charted data using a standardized form, and discrepancies were resolved by consensus. Vaccination status, circulating SARS-CoV-2 variant, metabolic measures such as insulin resistance, and longitudinal neurologic outcomes were recorded when reported; however, these variables were too inconsistently available for comparative synthesis.

### 2.7. Descriptive Synthesis and Presentation

No meta-analysis, pooled prevalence calculation, or formal regional-versus-global effect estimate was performed. Country counts were derived directly from the 27 included records. Symptom, laboratory, cardiovascular, treatment, and outcome findings were charted and interpreted at study level. Because denominators, definitions, and clinical settings differed across studies, findings are described qualitatively wherever possible; any retained numerical values are study-specific and should not be interpreted as pooled regional estimates. External international literature is used only for clearly identified contextual discussion.

## 3. Results

### 3.1. Study Characteristics and Regional Distribution

A total of 27 original clinical studies met the eligibility criteria. The corpus was composed of case reports, case series, retrospective cohorts, and multicenter observational studies; no review articles were included as evidence. Most studies were retrospective and single-center, with sample sizes ranging from individual cases to approximately 100 children. The geographic distribution was uneven and concentrated in the UAE, Saudi Arabia, and Qatar, with fewer reports from Jordan and multinational regional collaborations [7,8,9,10,12,13,14,15].

### 3.2. Geographic Distribution of Included Studies

**Figure 2:**
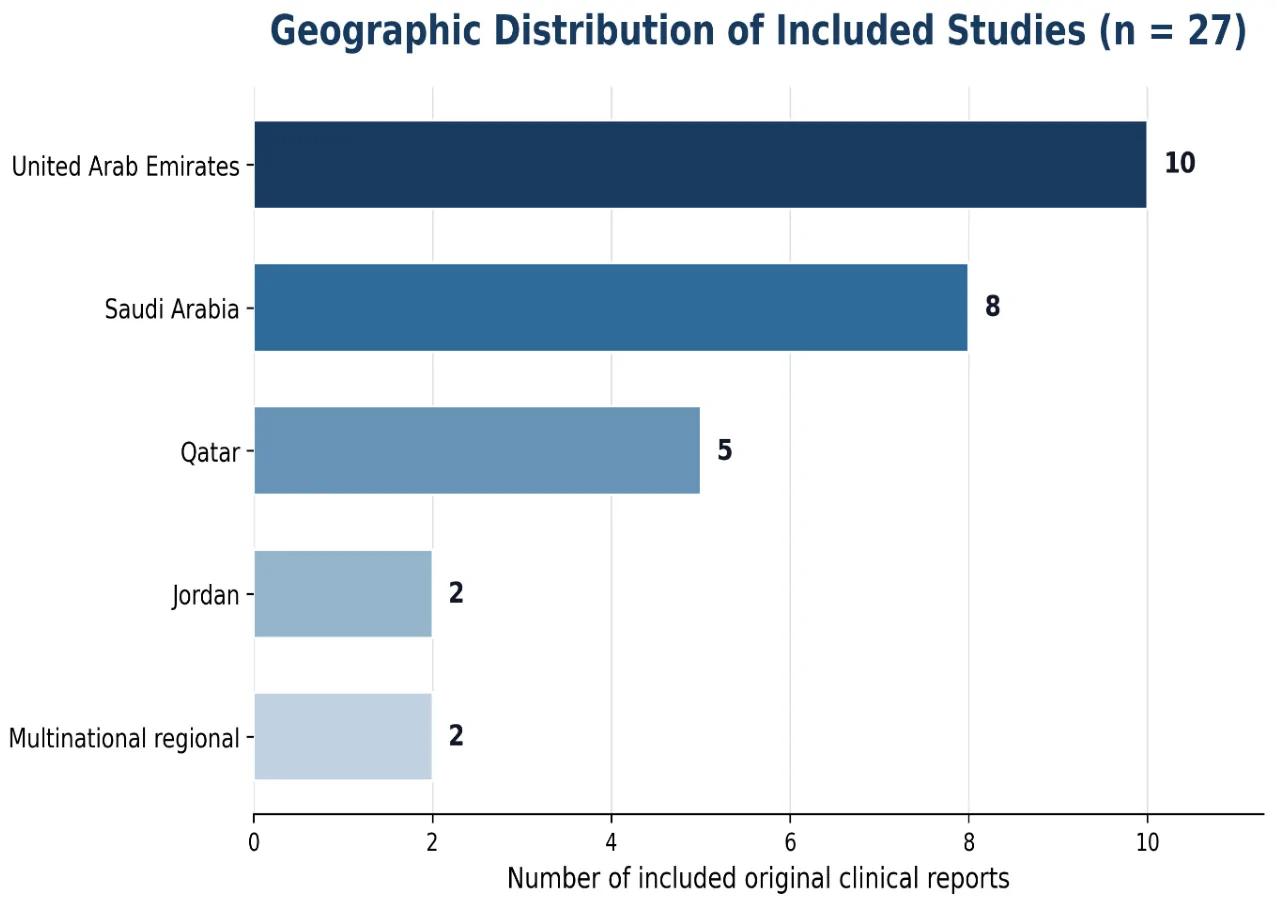
Study-level geographic distribution of the 27 included original clinical reports: UAE (n = 10), Saudi Arabia (n = 8), Qatar (n = 5), Jordan (n = 2), and multinational Middle Eastern collaborations (n = 2). Counts describe the evidence base and must not be interpreted as disease incidence or prevalence.

The 27 included studies showed a geographically uneven distribution across the Middle East. The largest number of studies originated from the United Arab Emirates (*n* = 10), followed by Saudi Arabia (*n* = 8), Qatar (*n* = 5), and Jordan (*n* = 2); two studies included multinational regional data. The geographic distribution of the included studies is presented in Figure 2.

### 3.3. Study Designs and Sample Sizes

The 27 included original clinical studies comprised case reports, case series, retrospective cohorts, and multicentre observational studies. Sample sizes ranged from individual patient reports to cohorts of approximately 100 children, reflecting substantial methodological heterogeneity across the available evidence.

### 3.4. Demographic and Clinical Presentation

MIS-C patients across the included cohorts were substantially older than typical KD populations. Median ages ranged from 6 to 10 years; adolescents were represented in most cohorts, contrasting sharply with KD, in which over 80% of cases occur before age five [7,14,16,17]. Slight male predominance was observed in most cohorts, consistent with global MIS-C data [1,2,16].

Fever was near-universal (reported in >95% of patients across cohorts) and typically prolonged, lasting more than 5 days in most reported cases [6,7,8,9,10,12,13,14,15].

Gastrointestinal symptoms, including abdominal pain, vomiting, and diarrhea, were commonly reported across the included studies, although their frequency varied according to study population, clinical setting, and reporting method [2,7,8,9,14,17,18].

Mucocutaneous manifestations—including rash, conjunctival injection, oral mucosal changes, and extremity swelling—were reported with varying frequency across the included studies [7,8,9,10,14,18].

Hemodynamic instability and shock were reported particularly in PICU-enriched cohorts, and several studies described the need for vasoactive support [1,2,8,9,19]. Respiratory involvement ranged from mild tachypnea to acute respiratory distress requiring mechanical ventilation but was generally less prominent than cardiovascular and gastrointestinal manifestations [19].

### 3.5. Contextual Illustration of MIS-C and Classic Kawasaki Disease Features

**Figure 3:**
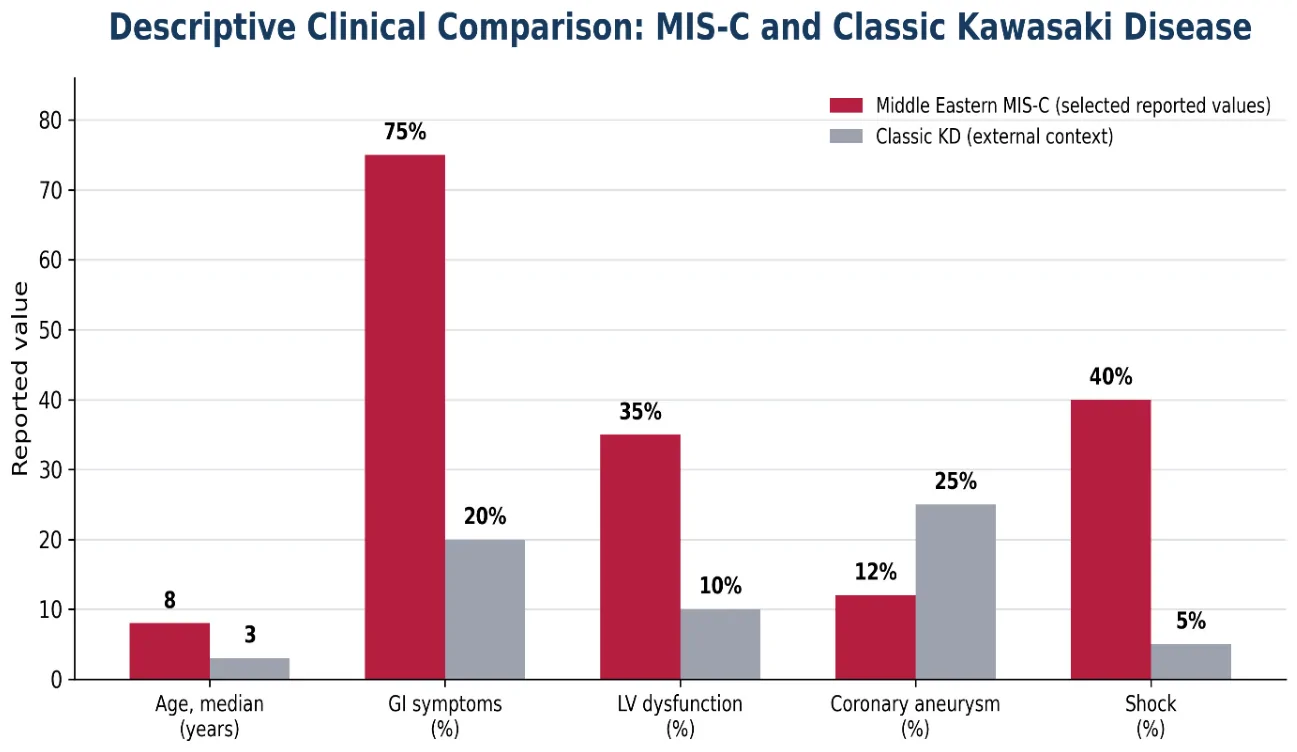
Contextual illustration of selected clinical features reported in included Middle Eastern MIS-C studies and external classic Kawasaki disease literature. Values were drawn from publications with differing populations, definitions, and denominators; they were not pooled or statistically compared and should not be interpreted as prevalence estimates or a formal comparative analysis.

Selected reported values from the included Middle Eastern MIS-C studies were descriptively compared with established features of classic Kawasaki disease (KD). MIS-C was associated with an older median age and higher reported frequencies of gastrointestinal symptoms, left ventricular dysfunction, and shock, whereas coronary aneurysms were more frequently reported in classic KD. These values are presented for descriptive context only and do not represent pooled estimates or a direct comparative analysis (Figure 3).

### 3.6. Laboratory Findings

Laboratory findings across included studies indicated intense systemic inflammation, hematological derangement, and coagulation activation [1,2,6,7,8,9,10,12,13,14,15,16].

Common findings included:

- Markedly elevated CRP, ESR, and ferritin in almost all patients.
- Lymphopenia and neutrophilia, contrasting with the leukocytosis and relative lymphocytosis of subacute KD [10,13,16].
- Thrombocytopenia in the acute phase, with occasional reactive thrombocytosis during recovery [16].
- Elevated D-dimer and fibrinogen, reflecting coagulation activation and thrombotic risk [16].
- Elevated cardiac biomarkers (troponin and BNP/NT-proBNP) were reported in several included studies and were observed alongside left ventricular dysfunction and shock [8,10,12,14,18,19].

In several regional cohorts, peak CRP and ferritin values were notably elevated, though quantitative comparison with international series is constrained by heterogeneous laboratory thresholds and case definitions across studies [12,13,14].

### 3.7. Laboratory Profile of Middle Eastern MIS-C

**Figure 4:**
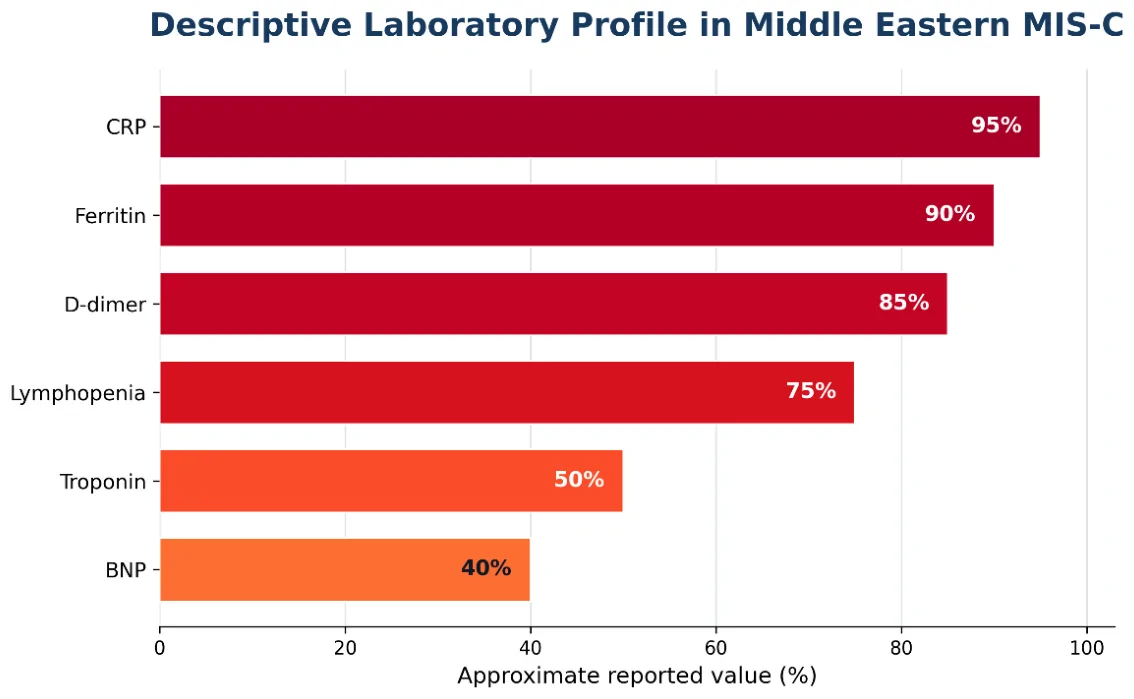
Descriptive heatmap of commonly reported laboratory abnormalities in selected Middle Eastern MIS-C studies. Color intensity represents approximate values reported across selected contributing publications with differing denominators; no pooled prevalence calculation was performed.

The included Middle Eastern studies consistently reported a marked inflammatory response in children with MIS-C. Elevated C-reactive protein and ferritin were among the most frequently reported laboratory abnormalities, together with elevated D-dimer and lymphopenia. Cardiac biomarker elevations, including troponin and B-type natriuretic peptide, were also reported. Selected approximate reported values are summarized descriptively in Figure 4 and should not be interpreted as pooled estimates.

### 3.8. Cardiovascular Involvement

Cardiac involvement was frequently described and was reported in association with greater clinical severity and need for intensive care across several cohorts [6,7,8,9,10,13,14,15,17,18,19].

Reported cardiovascular findings included:

- Left ventricular systolic dysfunction was reported in multiple regional studies; differences in case mix, echocardiographic definitions, and referral settings limited direct comparison across reports [8,9,10,14,18].
- Cardiogenic shock requiring vasoactive support was described particularly in PICU-enriched cohorts [8,9,17,18,19].
- Coronary artery dilatation or aneurysm was reported less frequently than ventricular dysfunction. Interpretation remains limited by differences in coronary definitions, imaging schedules, study populations, and denominators [10,11,14,18,20,21].
- Valvular regurgitation (particularly mitral) and pericardial effusion in a minority of cases [8,9,10,14].
- Arrhythmias and conduction abnormalitiesin a smaller subset [14].

Serial echocardiography, when reported, showed progressive improvement in LV function in the majority of patients. In most cohorts, ejection fraction was normalized by hospital discharge or within several weeks of commencing immunomodulatory therapy [6,7,8,9,10,13,17,18,22].

Notably, at least one UAE-based series documented delayed coronary artery dilation identified on echocardiographic follow-up despite normal initial imaging, underscoring the importance of structured cardiac surveillance beyond the acute admission period [10,14].

### 3.9. Cardiac Manifestations of MIS-C

**Figure 5:**
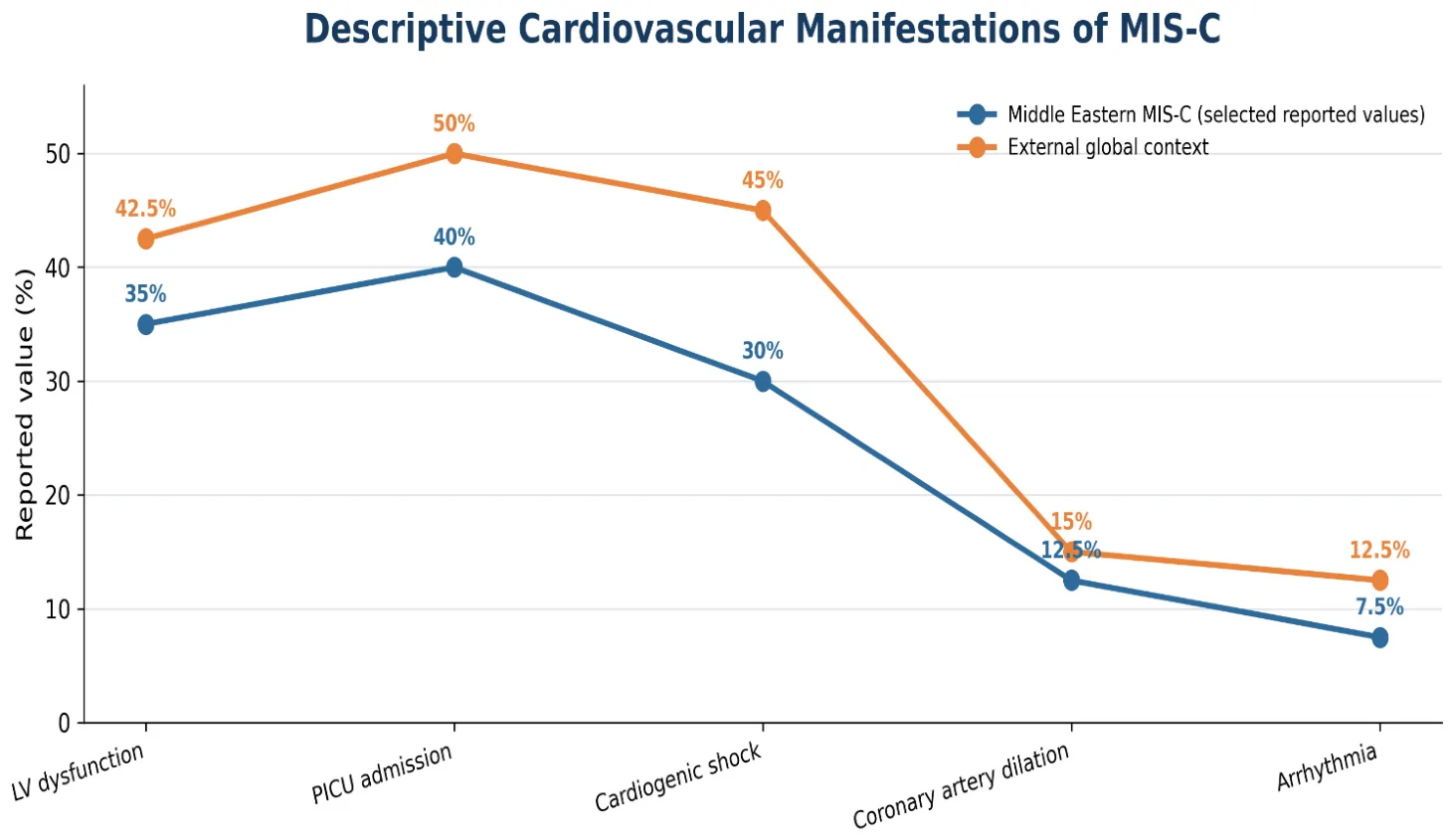
Descriptive visual summary of selected reported cardiovascular values in Middle Eastern MIS-C studies and external global contextual values. Denominators and study populations differed, and no pooled or formal comparative analysis was performed; the plotted values should not be interpreted as regional prevalence estimates.

Cardiovascular involvement was a prominent feature of MIS-C across the included Middle Eastern studies. Selected reported manifestations included left ventricular dysfunction, pediatric intensive care unit admission, cardiogenic shock, coronary artery dilatation, and arrhythmia. Figure 5 presents these selected Middle Eastern values alongside external global data for descriptive context only; the values are not pooled estimates or results of a direct comparative analysis.

### 3.10. Management Strategies

Management approaches across included studies generally included immunomodulatory therapy, antithrombotic agents, and hemodynamic or respiratory support, depending on clinical severity [11,16,20].

#### 3.10.1. Immunomodulatory Therapy

- **Intravenous Immunoglobulin (IVIG):** Most children received IVIG at 2 g/kg as first-line therapy, in line with KD practice and MIS-C guidance [7,8,9,10,12,14,16].
- **Corticosteroids:** Systemic glucocorticoids (e.g., methylprednisolone or prednisolone) were used in the majority of patients, either in combination with IVIG upfront or as early adjunctive therapy, aligning with American College of Rheumatology recommendations favoring IVIG plus steroids for most hospitalized MIS-C [16,20].
- **Biologic agents:** Anakinra (IL-1 receptor antagonist) and, less frequently, tocilizumab (IL-6 receptor antagonist) were reserved for refractory or severe cases with persistent shock, ongoing fever, or progression of cardiac dysfunction despite IVIG and steroids [8,9,12,14,23].

#### 3.10.2. Antithrombotic Therapy

- Low-dose aspirin (3–5 mg/kg/day, up to adult dosage) was commonly prescribed, especially in patients with coronary involvement or significantly elevated inflammatory markers [7,8,9,10,14,24].
- Low-molecular-weight heparin was used for thromboprophylaxis or treatment in patients with severe LV dysfunction, markedly elevated D-dimer, or additional thrombotic risk factors [24].

#### 3.10.3. Supportive Care

- Aggressive fluid resuscitation, vasoactive infusions, and respiratory support (including high-flow nasal cannula and mechanical ventilation) were standard in PICU patients [8,9,17,18,19].
- Broad-spectrum antibiotics were often initiated empirically until bacterial sepsis was excluded [25].
- Close multidisciplinary collaboration among pediatric infectious disease, cardiology, rheumatology, and critical care teams was common in larger centers [10,11,16,17,19,24,25,26].

### 3.11. Outcomes and Follow-Up

Short-term outcomes were generally favourable. Across cohorts, mortality was typically low (<2%), and most children experienced resolution of fever, normalisation of inflammatory markers, and recovery of LV function within weeks to months [1,2,6,7,8,9,10,12,13,14,18,27]. Length of hospital stay varied by severity and setting, but was often 7–14 days for uncomplicated cases and longer for those requiring PICU care.

Follow-up durations were heterogeneous. Some studies reported cardiology follow-up with repeat echocardiography at 2–6 weeks and again at 6–12 months for patients with significant initial cardiac involvement [10,12,13]. In these, most coronary dilations regressed, and LV function normalised, consistent with emerging international data from midterm cardiovascular cohorts [12,17,18,27]. However, very few Middle Eastern studies provided systematic data beyond 12 months, and advanced imaging (e.g., cardiac magnetic resonance imaging, strain echocardiography) was rarely reported, leaving potential subclinical myocardial fibrosis or subtle functional deficits largely unexplored.

## 4. Discussion

Twenty-seven studies from the UAE, Qatar, Saudi Arabia, Jordan, and multi-country Middle Eastern populations were included in this scoping review. The evidence base was dominated by retrospective, single-centre designs with modest sample sizes, which constrained the precision and generalisability of regional conclusions [10,11,12,20]. The available evidence nevertheless permitted cautious description of reported patterns and identification of important knowledge gaps.

### 4.1. Regional Phenotype in the Context of Global and Kawasaki Disease Data

Findings from the included Middle Eastern studies are discussed first, followed by clearly identified external evidence used only to provide clinical context. The broad clinical profile reported in the included regional studies was compatible with descriptions from large North American and European series [1,8,9], but the heterogeneous evidence does not support formal population-level comparison.

Across the included Middle Eastern cohorts, children with MIS-C were generally school-aged, with adolescents also represented. This differed descriptively from classic KD, which predominantly affects children younger than five years [2,5,6]. The Abuhammour et al. cohort from the UAE and Jordan reported a mean age of 6.7 years (SD 3.6) [26]. As external contextual evidence, an Iranian series reported a lower median age of 3 years [21]. Because the Iranian study was not one of the 27 included studies, it was not incorporated into the geographic distribution or regional synthesis. Differences between studies may reflect case ascertainment, referral patterns, or changing viral context, but population-level explanations cannot be inferred.

Within the included Middle Eastern studies, gastrointestinal complaints—abdominal pain, vomiting, and diarrhoea—were commonly reported [10,11,12,20,21,26]. External systematic reviews have also described gastrointestinal involvement as a prominent feature of MIS-C [14,16]. These separate evidence categories were not combined. Several included studies described abdominal imaging or surgical consultation for suspected appendicitis before MIS-C was considered [10,11], highlighting the importance of clinical awareness without permitting an estimate of comparative regional frequency.

Mucocutaneous features—including rash, conjunctival injection, oral changes, and extremity oedema—were described in the included regional studies, including the UAE/Jordan cohort [26]. External systematic reviews likewise reported these manifestations, although definitions and frequencies varied [14,16]. The apparent differences between regional and external reports may reflect documentation and case ascertainment and should not be interpreted as evidence of a distinct population-level phenotype.

The included regional studies frequently described myocardial dysfunction and the need for vasoactive support, whereas coronary artery dilatation or aneurysm was reported less often [1,8,9,13]. This descriptive pattern was also reported in external MIS-C literature [2,5,15,16], but the heterogeneous studies were not formally compared. One UAE-based series identified delayed coronary dilatation during follow-up after normal initial imaging, supporting continued cardiac surveillance beyond a single baseline echocardiogram.

Included Middle Eastern cohorts reported lymphopenia, neutrophilia, thrombocytopenia, elevated D-dimer, and raised troponin and BNP/NT-proBNP. External reports describe a broadly similar laboratory pattern and distinguish it from the typical haematological profile of classic KD [2,5,15,16]. Associations between cardiac biomarkers, PICU admission, and vasoactive support reported in regional and international studies remain observational and should not be interpreted as causal [10,23,24].

### 4.2. Treatment Approaches and Concordance with International Guidance

In the included Middle Eastern cohorts, management commonly involved IVIG at 2 g/kg and systemic corticosteroids [5,8,9]. Regional data were insufficient for an independent comparative-effectiveness assessment. As external contextual evidence, observational treatment studies from the United States and United Kingdom reported lower treatment-failure rates and faster haemodynamic recovery with combined IVIG and corticosteroids than with IVIG alone [8,9]. These external findings are discussed only to contextualise regional practice and were not included in the regional synthesis.

Within the included regional studies, escalation to anakinra or, less commonly, tocilizumab, was described for refractory shock, persistent fever, or worsening cardiac function despite first-line therapy [11,12,21,26,28,29,30]. External guidance describes similar indications, but the included studies rarely formalised escalation criteria; therefore, no regional treatment threshold or comparative effectiveness can be inferred.

Antithrombotic practice was more variable. Low-dose aspirin was commonly prescribed for patients with coronary involvement or pronounced inflammatory markers, while low-molecular-weight heparin was used selectively in those with severe ventricular dysfunction or markedly elevated D-dimer [11,12,21,26,28,30]. This heterogeneity likely reflects differences in local formulary availability, varying levels of comfort with paediatric anticoagulation, and the absence of randomised data to guide thromboprophylaxis in MIS-C. Until prospective regional studies document the incidence of thrombotic complications and the efficacy of preventive strategies, antithrombotic practice will remain empirical.

### 4.3. Identifying Children at Risk for Severe Disease

External contextual studies, rather than the included Middle Eastern corpus alone, provided most quantitative evidence on predictors of severe MIS-C. These studies reported associations of older age, elevated ferritin, lymphopenia, hypoalbuminaemia, elevated CRP, and depressed LVEF with ICU admission or severe disease [19,22,23]. Because the findings arose from separate observational cohorts, they are presented as hypothesis-generating associations and not as validated regional predictors or causal effects.

Regional genetic studies provide a potentially important but preliminary contribution. Abuhammour et al. reported rare heterozygous variants in innate immune pathways in a selected cohort, with a higher requirement for repeat IVIG among variant carriers [26], and a Kuwaiti study identified candidate monogenic susceptibility factors [28]. Because the samples were small, selected, and not replicated using comparable methods across multiple ancestries, the findings should not be interpreted as proof of a distinctive Arabian Peninsula genetic architecture or used to guide treatment outside research settings.

One multicenter study associated CNS involvement and fever above 39.5 °C with severe disease [28]. The finding may inform clinical vigilance but does not constitute a validated prognostic score. Prospective validation would require a prespecified cohort, standardized predictors and outcomes, derivation and external-validation samples, calibration and discrimination analyses, and comparison with existing severity assessment. Until then, immunomodulation should be initiated for diagnosed MIS-C according to clinical severity and established guidance, not administered prophylactically on the basis of fever and neurologic symptoms alone [6,31].

### 4.4. Biologic Agents: Balancing Efficacy Against Safety

External contextual safety evidence for anakinra and tocilizumab was derived primarily from paediatric rheumatology studies, prescribing information, and international guidance rather than from the included Middle Eastern MIS-C studies [31,32,33,34,35]. Injection-site reactions were commonly reported with anakinra, while serious infections were uncommon in the cited paediatric rheumatology data [32]. The short half-life of anakinra may be clinically useful when treatment must be discontinued [34], but these indirect data should not be interpreted as a regional MIS-C safety estimate.

These safety data originate almost entirely from chronic paediatric rheumatological conditions. In MIS-C, where biologic exposure is typically brief—days rather than months—the risk profile may differ. Prospective pharmacovigilance data specific to MIS-C are needed to inform evidence-based selection between these agents.

### 4.5. The Unresolved Question of Long-Term Cardiovascular Health

The included Middle Eastern MIS-C studies generally described recovery of left ventricular function within weeks and regression of many coronary abnormalities during follow-up [10,11,12,17]. Separately, external mid-term evidence, including a meta-analysis of 84 studies, described resolution of most cardiovascular abnormalities by six to nine months [27]. The external findings are presented only for context and were not combined with the regional data.

External non-Middle Eastern follow-up studies have reported persistent cardiac MRI abnormalities, reduced exercise capacity, and occasional longer-term coronary abnormalities despite apparent echocardiographic recovery [27,36]. These observations do not constitute findings of the present regional synthesis, but they identify outcomes that should be investigated prospectively in Middle Eastern cohorts.

In the included Middle Eastern literature, follow-up rarely extended beyond 12 months, and advanced imaging such as cardiac MRI or speckle-tracking echocardiography was seldom reported. As external guidance, the AHA scientific statement recommends structured follow-up and selective cardiac MRI after moderate-to-severe LV dysfunction [37]. This recommendation is presented as contextual guidance rather than as a finding derived from the included studies.

Neurocognitive outcomes, psychosocial functioning, and health-related quality of life after MIS-C have received almost no attention in the Middle Eastern literature—or, for that matter, in the global literature. As the acute phase of the pandemic recedes, these domains deserve dedicated investigation.

### 4.6. Strengths and Limitations

This review applied the Arksey and O’Malley framework with JBI guidance and followed PRISMA-ScR reporting. Independent screening and data charting were undertaken. Its principal contribution is the mapping of reported evidence and research gaps across a geographically uneven corpus; it does not provide pooled regional estimates or comparative effect measures.

The limitations are substantial. Most included studies were retrospective and single-center, case definitions evolved during the pandemic, laboratory and echocardiographic protocols were not standardized, and follow-up was inconsistent. Publication and selection bias may have favored severe or unusual cases, particularly in PICU-based cohorts. Country coverage was uneven, and duplicate or overlapping populations could not always be excluded with certainty. Vaccination status and circulating SARS-CoV-2 variants were inconsistently reported, preventing assessment of temporal or variant-related differences. Metabolic measures such as insulin resistance were rarely available. These limitations constrain cross-study comparisons and preclude causal or population-wide regional conclusions.

Although PubMed/MEDLINE, Scopus, Web of Science, and the WHO COVID-19 database were searched, Embase, dedicated regional indexes, grey literature, and conference proceedings were not systematically searched. Relevant evidence may therefore have been missed. Database-specific syntax was adapted from a common search logic, and the review protocol was not prospectively registered. Records from the four databases were merged before deduplication; although the documented combined database total and study-selection pathway were retained, the archived source-specific export logs and individual database retrieval counts were not available for source-level reconstruction.

## 5. Conclusions

The reported Middle Eastern evidence suggests that MIS-C often affects school-aged children and includes gastrointestinal, mucocutaneous, and myocardial manifestations. These descriptive patterns are broadly compatible with international reports, but the regional corpus is too small, heterogeneous, and geographically concentrated to support precise regional prevalence estimates or definitive comparisons with classic Kawasaki disease or non-Middle Eastern populations.

IVIG and corticosteroids were commonly used, with biologic escalation reported for refractory disease. Favorable short-term outcomes were described, but treatment effectiveness cannot be inferred from these uncontrolled, predominantly retrospective data.

Candidate immune-related variants reported in selected Middle Eastern cohorts are hypothesis-generating. Replication in larger, ancestry-aware, multicenter studies is required before any population-specific susceptibility or treatment implication can be claimed.

The main contribution of this scoping review is to identify evidence gaps: prospective multicenter registries, standardized case definitions and outcome measures, transparent reporting of vaccination and viral-variant context, metabolic characterization, validated severity-prediction studies, and structured long-term cardiac and neurodevelopmental follow-up.

## Data Availability

The data supporting the findings of this study are available within the article and its referenced sources. No new datasets were generated during the current study.

## Supplementary Materials

The following supporting information can be downloaded at https://www.mdpi.com/article/10.3390/medicina62081600/s1: PRISMA extension for scoping reviews (PRISMA-ScR) checklist. Reference [38] is cited in the Supplementary Materials.

## Abbreviations

MIS-C	Multisystem Inflammatory Syndrome in Children
KD	Kawasaki Disease
SARS-CoV-2	Severe Acute Respiratory Syndrome Coronavirus 2
PIMS-TS	Pediatric Inflammatory Multisystem Syndrome Temporally Associated with SARS-CoV-2
PICU	Pediatric Intensive Care Unit
IVIG	Intravenous Immunoglobulin
LV	Left Ventricular
BNP	B-type Natriuretic Peptide
CRP	C-Reactive Protein
ESR	Erythrocyte Sedimentation Rate
CDC	Centers for Disease Control and Prevention
WHO	World Health Organization
JBI	Joanna Briggs Institute
PRISMA-ScR	Preferred Reporting Items for Systematic Reviews and Meta-Analyses Extension for Scoping Reviews
